# Bio-fabrication of biologically active copper nanocomposite

**DOI:** 10.1016/j.heliyon.2024.e40202

**Published:** 2024-11-09

**Authors:** Nehal Eid, Hesham R. El-Seedi, Hanan B. Ahmed, Hossam E. Emam

**Affiliations:** a Department of Chemistry, Faculty of Science, Menoufia University, 32512, Shebin El-Kom, Egypt; b International Joint Research Laboratory of Intelligent Agriculture and Agri-products Processing (Jiangsu Education Department), Zhenjiang, 212013, China; c Chemistry Department, Faculty of Science, Helwan University, Ain-Helwan, Cairo, 11795, Egypt; d Department of Pretreatment and Finishing of Cellulosic based Textiles, Textile Research and Technology Institute, National Research Centre, Scopus Affiliation ID 60014618, 33 EL Buhouth St., Dokki, Giza, 12622, Egypt

**Keywords:** Sidr honey, Cu, Bio-fabrication, Antimicrobial potentiality, Anticancer performance, Anti-inflammation action

## Abstract

Green routes for nucleation of nanostructures are recently applied for the biomedical applications, due to their affinity and feasibility. For instance, copper nanostructures are applicable for inhibition of microbial infections and ulcers. Hereby, this work is aimed at investigation for the possible contribution of Sidr honey (SH) in the formation of copper based-nanocomposites (Cu@SH). The overall results affirmed that, under the optimal experimental conditions, spherical shaped Cu@SH nanocomposites with quite small particle size of 16.2–30.1 nm were successfully clustered. The bio-fabricated Cu@SH nanocomposites showed superior antimicrobial action against all of the examined strains, whereas, the most potent inhibition was obtained against *St. aureus* (11 mm), *E. Coli* (17 mm) & *C. albicans* (14 mm). The nanocomposite that was prepared with higher concentration of copper precursor is exhibited with the highest percentage of cancer cell inhibition (77 %) and highest anti-inflammation action (NO percentage 89 %). In summarization, Cu@SH nanocomposites showed high affinity to be concurrently applicable as therapeutic drugs for the microbial infectious, cancer and inflammation diseases.

## Graphical abstract

**Figure:**
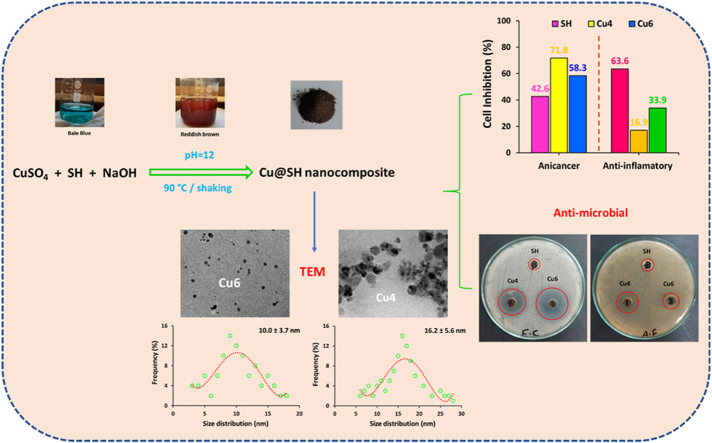

## 1 Introduction

In the last decade, nanotechnology found to be encompassed many fields, including the environmental protection, the wastewater treatment, the chemical industrialization, production of solar cells, microbicidal therapeutics, textile industry, dyes production, and healthcare [[1], [2], [3]]. According to literature, nanotechnology engages within these applications by time. It becomes well-known that the main characteristics of any material could be significantly altered with its nucleation in nano-size, attributing to "the quantum size effects". Metallic based nanocomposites, in particular, show superior photoluminescent properties, thermal stability, chemical reactivity, and conductivity compared to their original metallic forms. Nano-sized composites are found to be excellently applicable in many purposes owing to as-the mentioned characteristics [4,5].

Green synthetic routes have recently shown to be the most favorable methods of synthesis [6]. Green nano-synthesis promotes the successive clustering of nanostructures in environmentally safe pathway for both human beings and the surrounding environment. Through green nano-synthesis, no toxic chemical byproducts are discharged to the surrounding environment and, instead, the non-harmful substrates are exploited for nucleation. Microorganisms such as "bacteria, yeast and mold", plant and animal extracts were numerously exploited for green synthesis of nanostructures, whereas, such synthetic precursors are identified as eco-safe, non-toxic, cost effective and are active under the mild conditions [[7], [8], [9], [10]]. Moreover, in agreement to the green chemistry aspects, various plant extracts, polysaccharides [[11], [12], [13], [14]], algae, fungi, bacteria, yeasts and marines [15] were reported to be successfully applicable as substrates for various types of nanostructures.

Natural resources were reported with remarkable characters, due to the rich content of bioactive ingredients such as "flavonoids", "phenols", "terpenoids", and "steroids". Such components act in the biogenic reduction of metal ions without the requirement of any additional chemical reagents. Moreover, they can superiorly act as capping agents for controllable crystal growth, morphological and size regulation, and decrement of coagulations for nanoparticles. It was also mentioned that, the biosynthesized nanostructures are found to be more stable than that nucleated via the physico-chemical techniques, making it superiorly exploitable for wide scaled applications such as "catalysis", "bio-sensing", "pharmaceutic", "biomedicine" and "drug delivery" [15,16]. Sidr honey is one of the widely known uni-floral honey that is obtained from "*Ziziphus spinachristi* L″ or "Christ's Thorn Jujube" in the desert of "Egypt", "Saudi Arabian", and "Pakistan". Sidr honey is known to exhibit antioxidation and antimicrobial effects, so as it could be applicable in wound healing, and it also exhibits antidiabetic potency. The antimicrobial performance was evident when Sidr honey is found to prevent the growth of pathogenic strains such as *"Staphylococcus aureus", "Streptococcus mutans", "Klebsiella pneumoniae", "Escherichia coli"* and *"Pseudomonas aeruginosa"* in in vitro sets [17].

On the other hand, copper nanoparticles (CuNPs) were reported to be advantageous with catalytic, optical, and electrical properties, to be successfully applicable in the electricity sector, attributing to their conductivity properties. Additionally, they were found to be used in industry, as " lubricator" and "catalyst" [[18], [19], [20]]. Copper (II) oxide nanoparticles (CuO NPs) were also characterized with semi-conductivity, to be often applied in catalysis, gas sensing, and manufacturing of the photovoltaic cells. Owing to its electronic and magnetic properties, CuO was applied as one of the main ingredients of the high temperature superconductive components, to be world widely applicable for such purpose [21]. Etefagh et al. succeeded in the preparation of a sensor of CuO nanoparticles (CuONPs) in the form of nanolayers. CuONPs were ingrained using "the sol–gel technique", and their nanolayers were formed via "spray pyrolysis technique" [22]. Phiwdang et al., investigated the "precipitation technique" for synthesis of nanostructures where the structural characters of CuONPs were ingrained [23]. Khashan et al., also tested the ablation duration and the laser energy on the properties of the clustered CuONPs via "laser ablation technique" in liquid. Bactericidal activities of these CuONPs with or without amoxicillin on the "Gram-negative" and "Gram-positive" bacteria cultures were also evaluated [24].

Practically, the point of novelty is to demonstrate a green synthesis and investigating the affinity of Egyptian sidr honey (SH) to act as substrate for successive clustering of copper-based nanocomposite, abbreviated as Cu@SH, in order to be applicable as antimicrobial/therapeutic drug. Sidr honey played a dual role as reducer and stabilizer for the required nanostructures, without adding any of extra chemical ingredients. NMR spectral mapping data were examined in order to confirm the composition of Cu@SH nanocomposite. The successive nucleation of Cu@SH nanocomposites was monitored via UV–visible spectral data, Transmission Electron Microscope (TEM), Zeta sizer, X-ray diffraction (XRD) and X-ray photoelectron spectroscopy (XPS). The obtained Cu@SH nanocomposites were examined as antimicrobial therapeutic drug against different microbial pathogenic strains. In addition, its propensity to act as anticancer and anti-inflammatory reagents was also approved.

## 2 Experimental

### 2.1 Materials and chemicals

"Sidr honey (SH)" samples were collected from "Bee Research Center" (Al Qanater Charity; Egypt). Copper Sulfate (CuSO_4_.5H_2_O, ≥98 %), and "Sodium hydroxide" (NaOH, 99 % from Sigma Aldrich) were exploited in the experimental work as received.

### 2.2 Cu@SH nanocomposite clustering

The experimental conditions that were proceeded for preparing six samples of Cu@SH nanocomposites were clarified in Table 1. In a typical experiment, specific volume of diluted Sidr Honey (20 %) was transferred to 50 mL container and 20 mL of NaOH solution (1 M) was added. The temperature of reaction vessel was adjusted under continuous stirring (700–800 rpm) and then certain concentration of CuSO_4_.5H_2_O was added. The redox reaction between Sidr Honey and Cu was proceeded at 90 °C. For following up the reaction, samples were withdrawn at different time intervals (5, 30, 60 min) to stop the reduction reaction. The samples were labeled as Cu1- Cu6 according to the experimental conditions. For samples of Cu5, Cu6, NaOH was added from the beginning with the Sidr honey.

**Table 1:** Biofabrication details for Cu@SH nanocomposite samples.

**Samples**	**SH (mL)**	**CuSO**_**4**_**(1 mM, mL)**	**Time (min)**	**Particle size (nm)**		**pdI**	**Zeta potential (mV)**
				**TEM**	**Zetasizer**		
Cu1^a^	10	10	0	–	122.4	0.65	−24.7
Cu2^a^	10	10	5	–	91.3	0.46	−11.1
Cu3^a^	10	10	30	–	50.7	0.31	−25.6
Cu4^a^	10	10	60	16.2	68.1	0.29	−12.8
Cu5^b^	10	10	60	30.1	164.2	0.31	−15.6
Cu6^b^	20	5	60	10.0	25.4	0.41	−28.5

PH = 12, Temperature = 90 **°**C.

a NaOH added secondly after the temperature reached 90 °C.

b NaOH added from the beginning with Sidr honey (SH).

### 2.3 Analysis and characterization

^1^H NMR spectral data were estimated via "Jeol EX-400 spectrometer: 400 MHz", while HSQC spectra were collected at 298 K on a Bruker 600 MHz (TCI CRPHe TR-1H and 19F/13C/15N 5 mm-EZ CryoProbe) spectrometer. The chemical shift was referenced to the solvent band for CD3OD at "*δ* ^1^H at 3.3 and 4.8 ppm & *δ* 13 C at 49.00 ppm, respectively". The successive nucleation of Cu@SH nanocomposite was monitored via UV–Visible spectrophotometer (T80 UV/VIS, from PG Instruments Ltd, Japan), whereas, the absorption spectral maps for the prepared samples were collected at 250–750 nm using 2 nm intervals. SH and copper sulfate solutions were examined as blank samples.

"High Resolution Transmission Electron Microscope, JEOL-JEM-1200 from Japan" was applied for the characterization of the topography and for estimation of the size distribution for all of the examined Cu@SH nanocomposites. Nanocomposite colloid was uploaded over "400 mesh carbon-coated copper gride", then was kept at room temperature for vaporization to be sequentially connected to microscopic examination. The size average was estimated from the obtained micro-images using "4 pi analysis software from USA". The size average was evaluated for fifty particles at different selected areas in the considered image. Zetasizer was used for estimating the size distribution, mean size and index of poly-dispersity (PdI) of the prepared Cu@SH nanocomposites. The measurements were performed via the dynamic light scattering technique with "Malvern Zetasizer Nano ZS, from Malvern Instruments Ltd – UK".

Diffraction of the prepared Cu@SH nanocomposites were analyzed at room temperature using "X'PertPRO PANalytical diffractometer, from Malvern Panalytical", whereas, the diffraction angle (2θ) was estimated at 5°–80° using "monochromatic PC computer with the programs PROFIT, Cu Kα X-radiation at 40 kV and λ = 1.5406 Å". Moreover, the synthesized Cu@SH nanocomposites were also analyzed with "X-ray photoelectron spectroscopy (XPS), X-Ray – FG ON (400 μm)". The examined samples were firstly neutralized with low energy electrons (0.1 eV), via "magnetic and electrostatic lenses, as hybrid mode, scanning times of 10–22, 400 μm spot size, 9–10 mbar pressure and full spectra pass energy 50–200 e.v" were all exploited. The photoelectrons excitation was proceeded via "monochromatic Alpha radiation".

### 2.4 Antimicrobial performance

In vitro antimicrobial performance for the examined samples was approved, while, the pathogenic strains used in the current approach were Gram-positive bacterial species (*Staphylococcus Aureus* ATCC-6538), Gram-negative bacterial species (*Escherichia Coli* ATCC-25922) and two fungal strains namely (*Candida Albicans* ATCC-10231, *Aspergillus Fumigatus* ATCC-1022). The as-chosen microbial strains were obtained from "American Type Culture Collection" (ATCC, Rockville MD, USA), and "Northern Utilization Research and Development Division", "United State Department of Agriculture, Peoria, Illinois, USA (NRRL)". The microbial strains were revived for examination by sub-culture in "the fresh nutrient agar (NA) medium (Merck, Darmstadt, Germany)" for one day before starting the evaluation process. Whereas, fungal strains were cultured on "potato dextrose agar (PDA) (Lab M., Bury, Lancashire, UK)" for one week at 28 °C before starting the experiments.

Stock media were maintained at 4 °C in "nutrient agar and potato dextrose agar". The active media were prepared for evaluation via transferring lapful of pathogenic cells from the stock media to the test tube of " Mueller-Hinton broth (MHB) (Lab M Limited, Bury, Lancashire, UK)" for bacterial species and "Sabouraud dextrose broth (SDB) (Lab M., Bury, Lancashire, UK)" for fungal strains to be incubated at 37 °C and 25 °C, respectively without any agitation for one day. To 5 mL of "MHB" or "SDB", 0.2 mL of microbial colloid was inoculated to be incubated till turbidity equal to "the standard 0.5 McFarland solution" at 625 nm (A = 0.08 to 0.1) that is equal to 1.5 x 10^8^ cfu/mL.

Agar diffusion disc method as reported by Perez et al. [25] was applied for the evaluation of in vitro antimicrobial action of the samples. 0.1 mL liquor from 18 h broth medium of the above-mentioned pathogenic strains [26] was dispensed into the sterile Petri dish that was formerly labeled with the examined microbial strain. The molten sterile medium "Muller-Hinton" was aseptically dropped into the dishes and gently rotated for the microbe to be homogeneously dispersed in media. The microbe screening bio-evaluation was performed via agar well-diffusion technique reported by Jorgensen and Turnidge [27] using "Mueller-Hinton agar (Lab M Limited, Bury, Lancashire, UK)". The agar dishes were left to be solidified, then 11 wells (9 mm) were cut by sterile "Cork borer" and 100 μL of standard and examined samples were added in each well, then the dishes were promoted to be diffused for 120 min at 4 °C. The procedure was performed three times and the average was evaluated. All the prepared dishes were allowed to be incubated at 37 °C for one day & 28–30 °C for two days in case of bacterial & fungal strains, respectively. the diameter of clear zone around the well was evaluated in millimeters [28]. Standard microbial antibiotics as "Tetracycline (30 μg)" and antifungal drug as "Cycloheximide (50 μg)" were used as references i.e., as positive control, for the chosen bacterial and fungal strains, respectively.

#### 2.4.1 Cell proliferative assay and anticancer performance

The cell proliferative assay is performed for monitoring the change in physiological and physical properties of the cells that were exposed to the extracellular stimulation [29]. "Sulforhodamine B (SRB) assay" is a colorimetric test that mainly depends on the optical activity of SRB dye that is chemically interacted with the basic amino acids in the cell proteins. Whereas, the total mass of proteins is estimated via counting the cells. In this approach, SRB assay was performed to affirm the anticancer potency of the nucleated Cu@SH nanocomposite and to monitor their anti-cancer performance against "lung cancer cell (A-549)". A-549 cell line was collected in "Nawah Scientific Inc., (Mokatam, Cairo, Egypt)". The collected cells were allowed to grow in "DMEM medium supplemented with 100 mg/mL of streptomycin, 100 units/mL of penicillin and 10 % of humidified/heat-inactivated fetal bovine serum and five percent of (v/v) CO_2_ atmosphere at 37 °C". Liquors of "100 μL cell (5x10^3 cells) were suspended in 96-well plates for incubation up to one day". The examined cells were mixed with another colloid of "100 μL medium containing the prepared nanocomposite with different concentrations". After 72 h, the examined cells were modified with "150 μL of 10 % trichloroacetic acid (TCA) to be incubated at 4 °C for 60 min". TCA was removed, whereas, the tested cells were washed for 5 times with water. Liquors of "70 μL SRB solution (0.4 % w/v) was added to transfer cells for another time of incubation in dark at ambient conditions for 10 min". Subsequently, the cells were washed for 3 times with "1 % CH_3_COOH for air-drying overnight". Eventually, 150 μL of TRIS (10 mM) was used to dissolve the complex of protein-SRB, for estimation of Absorbance at "540 nm via BMGLABTECH®- FLUOstar Omega microplate reader, Ortenberg, Germany".

#### 2.4.2 Anti-inflammation performance

In the current work, anti-inflammation performance of the as-prepared Cu@SH nanocomposite was examined according to Griess assay [30] that depends on the estimation of nitrous oxide "NO" percentage. Whereas, equal amounts of Griess drug and culture supernatant were mixed and incubated at the ambient condition for 10 min to give the colored diazonium salt and for the estimation of the absorbance at 540 nm on "Tecan Sunrise™ microplate reader" (Austria). NO Inhibition percentage of Cu@SH nanocomposite was detected according to "LPS-induced inflammatory groups", with normalizing for the cell viability estimated via "Alamar Blue™ reduction assay" [31]. The concentration that prevented 50 % of LPS-induced NO was statistically estimated on "GraphPad Prism software via the curve fitting to a non-linear regression modeling".

## 3 Results and discussion

**Fig. 1:**
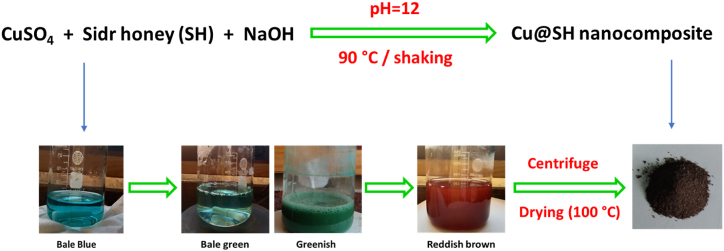
Biofabrication process of Cu@SH nanocomposite.

The current approach is aimed to the investigation of Egyptian sidr honey affinity as a substrate for synthesis of Cu@SH nanocomposite to be applied as antimicrobial, anti-inflammatory and anticancer agents. Fig. 1 represents a schematic diagram for the mechanism for nucleation of Cu@SH nanocomposite from Egyptian sidr honey. As reported in literature [32], SH contains "monosaccharides (maltotetraose, stachyose, maltose and galactobiose), flavonoids and terpenoids". The reducible groups within the main skeleton of SH (especially in sugars) were superiorly acted in clustering of Cu@SH nanocomposite. Mainly, the redox reaction is ascribed as the milestone in the preparation of Cu@SH nanocomposite.

Firstly, copper ions were preliminary adsorbed on the surface of SH building blocks. Whereas, the alcoholic groups (-CH_2_OH) in SH were reported to be oxidized, while copper ions were sequentially reduced to the atomic form, whereas, NaOH as a strong alkali is added to the reaction liquor in order to activate the SH functional groups to interact with metal ions for the successive clustering of NPs [[33], [34], [35], [36]]. The first nucleus of nano-copper acted as an active center for further reduction of the remaining copper ions [37,38]. The formed nanoclusters were protected by action of SH building units. The reaction conditions for synthesis of Cu@SH nanocomposites were tabulated in Table 1.

### 3.1 NMR analysis

**Fig. 2:**
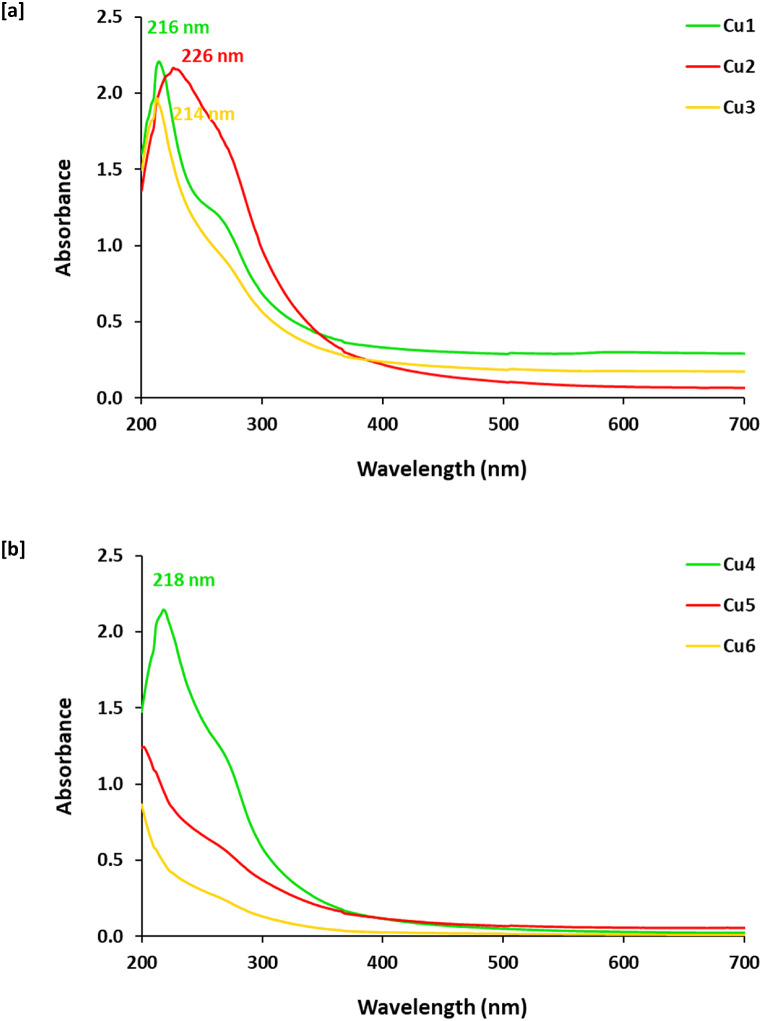
Absorbance spectra of the prepared samples.

In accordance to literature [32,39,40], the major components of sider honey (SH) are reported to be saccharides and organic acids as primary metabolites. Fig. S1a for Cu4 shows a significant band at *δ* 2.5–2.8 ppm corresponds to methyl group (CH_3_) and the significant bands of the methylene (CH_2_) at *δ* 3.5–4.5 ppm affirm the existence of sugars units. Also Fig. S1b for Cu6 shows a characteristic peak at *δ* 2.5–2.8 ppm corresponds to methyl group (CH_3_) and the significant bands of the methylene (CH_2_) and methine (CH) groups appear between *δ* 3.5–4.5 ppm to affirm the existence of sugars units. The detection of anomeric protons at *δ* 5.18 ppm (d, *J* = 3.4 Hz) ppm supports the presence of *α*-glucose units [41]. On the other hand, Figs. S1c and d shows ^13^CNMR spectral data. Fig. S1c declares that, for Cu4, the detected peaks at 10–40 ppm, 69–73 ppm and 130 ppm are corresponding to simple sp^3^ carbons, O-CH of alcohol and sp^2^ carbon attached to an electronegative atom (oxygen or nitrogen) or Cβ carbon conjugated with a carbonyl group, respectively. Similarly, Fig. 2d shows that, the spectrum of Cu6 is shown with significant peaks for sp3 carbons, sp2 carbons, sp carbons, -O-CH, sp^2^ carbon attached to an electronegative atom (oxygen or nitrogen) or Cβ carbon conjugated with a carbonyl group, carboxyl carbons and aldehyde carbons, at 12–14 ppm, 19–23 ppm, 33–41 ppm, 62–69 ppm, 81–90 ppm, 146 ppm, 169–177 ppm and 181–188 ppm, respectively [42]. Taken together, detection of aldehyde carbons and/or carboxyl carbons can approve the hypothesized mechanism of redox reaction between SH ingredients and copper ions for successive ingraining of the as-desirable Cu@SH nanocomposites.

### 3.2 UV–vis spectral maps

The change of reaction liquor solution from pale yellow to dark yellow then to reddish brown color is a primer visual signing for successive clustering of Cu@SH nanocomposites. The captured photos of the prepared samples and the absorbance spectral data were presented in Fig. 2. UV–visible peak is reported to be significantly affected by different key factors such as particle size of the dispersible nano-objects and the dielectric constant of the colloidal medium [13,43,44]. From the plotted data it could be depicted that, spectra of Cu1, Cu2, Cu3, Cu4 have a significant band around 214–226 nm, that is referred to the phenolic groups of SH. After 5 min the peak was shown with broadening, whereas, prolonging of reaction duration to 30 min resulted in decrement of band intensity. However, the peak disappeared in the spectra of Cu5 & Cu6 samples (Fig. 2b) attributed to, of the addition of sodium hydroxide to SH at the beginning of the procedure. The mechanism of action is thought to be the vigorous dissolution of SH ingredients leading to more accessible/reducible fragments.

### 3.3 Estimation of particle size

**Fig. 3:**
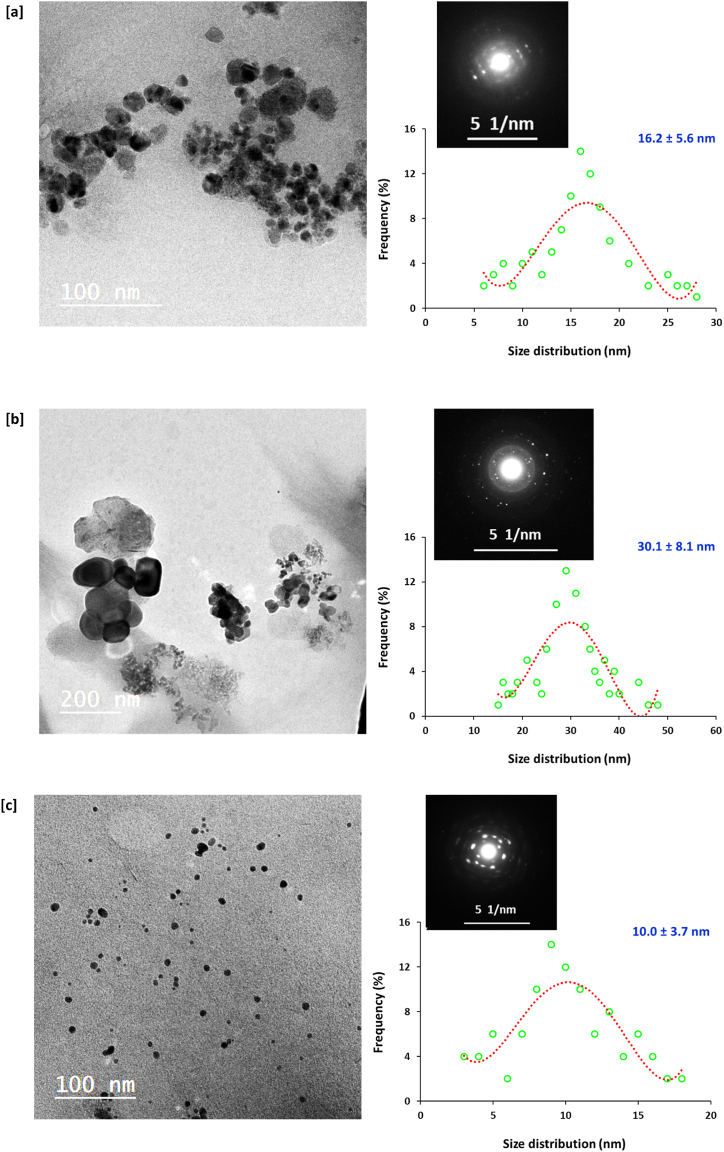
Transmission microscopic images, size distribution and diffraction patterns for the prepared Cu@SH nanocomposites; **[a]** Cu4, **[b]** Cu5 and **[c]** Cu6.

The topography, geometrical features and size distribution of nanocomposites were measured using TEM micro-images. Fig. 3 & Table 1 displays the spherical shaped/well-dispersed nanostructures where the size distribution was in the range of 10.0–30.1 nm depending on the experimental conditions. Whereas, Cu@SH sample (Cu6) prepared with the lowest concentration of metal precursor (5 mM/mL) exhibited the smallest particles size of 10.0 nm. Whereas, addition of sodium hydroxide at the beginning with duplication the concentration of metal precursor (Cu5) leaded to the nucleation of the largest sized particles (30.1 nm), that could be attributed to the effect of such mentioned experimental conditions in vigorous degradation of SH ingredients to produce fragments with lower affinity for stabilizing the as-nucleated nanostructures, resulting in their agglomeration, to be estimated with largest size. Additionally, the analysis of the diffraction pattern in each examined sample declares that, all the examined Cu@SH nanocomposites were shown with polycrystalline structure as the nano-shell construction was noted with the fine spots. Diffraction of the chosen area (SEAD) with the as-shown arcs confirms the prepared Cu@SH nanocomposites to be exhibited with highly-regulated crystalline structure.

**Fig. 4:**
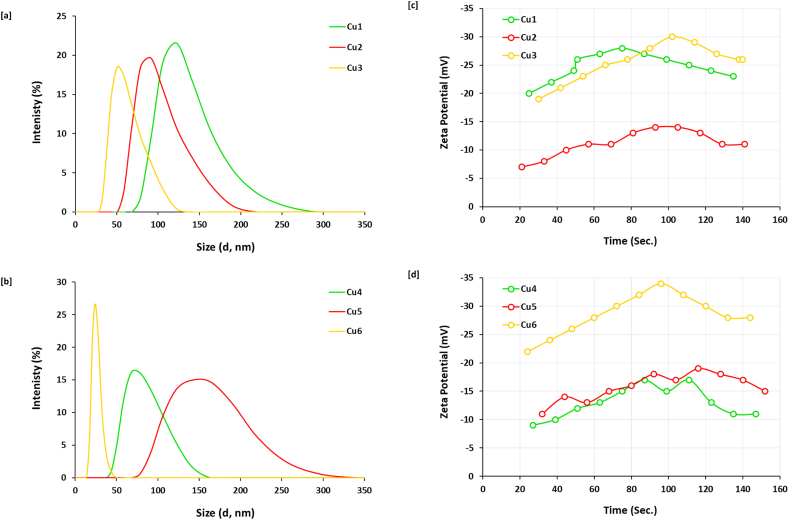
Particle size analysis and zeta potential from Zetasizer for the prepared nanocomposite; **[a, b]** particle size and **[c, d]** zeta potential.

Analysis of Zetasizer data was performed for more confirmation of the estimated size average for all of the prepared samples, whereas the evaluated results were plotted in Fig. 4 & Table 1. The data reveals that, Cu5 is estimated with the largest particle size of 164.2 nm, whereas, Cu6 is shown with the smallest particle size of 25.4 nm. Moreover, prolonging of the reaction duration from zero (Cu1, 122.4 nm), to 30 min (Cu3, 50.7 nm) resulted in controllable particle size of the nucleated nanocomposites, however longer time of reaction resulted in clustering of Cu@SH nanocomposite (Cu4) with much enlarged particle size of 68.1 nm. All of the illustrated data of size estimation is in harmony with that of absorbance spectral data. The clustered Cu@SH nanocomposites were shown with uniform as seen from Fig. 4 due to their controllable size and topography. The poly-dispersity index (PdI) was detected via the dynamic light scattering to detect the dispersion homogeneity for the as-prepared nanocomposites. In accordance to the data in Table 1, the obtained Cu@SH nanocomposites were characterized with a good stabilization via estimating PdI values in range of 0.29–0.65, whatever the experimental conditions of nucleation process. In accordance to literature, the best typical stabilization for nano-colloid was realized by estimation of PdI value near of 0.3 [33,37] which is estimated for Cu3, Cu4, Cu5 & Cu6.

On the other hand, Fig. 4c and d shows the plotted data of the Zeta-potential for all of the prepared Cu@SH samples. Zeta-potential is a key parameter for identifying the degree of electrostatic repulsive forces between similarly charged moieties in colloidal solution. The larger is the estimated negatively value of zeta-potential, the higher is the electrostatic repulsive forces between colloidal particles. Meaning that, the higher negatively charged value, the more is the degree of stability of the nano-colloid [32]. In agreement to literature, the smaller negative value of zeta potential is assigned to the particle agglomeration due to weak van der Waals attraction forces, leading to weaker physical stabilization of the nucleated nanocomposite [45]. From the plotted data in Fig. 4c and d & Tables 1 and it could be declared that, Cu6 is estimated with the highest negatively charged value of zeta potential (−28.5 mV). Meaning that, mixing of sodium hydroxide with SH in the beginning of the experiment with metal precursor concentration of only 5 mM/mL, is the most preferable conditions for successive clustering of highly stabilized Cu@SH nanocomposite. Whereas, duplication of the concentration of metal precursor resulted in nucleation of Cu@SH nanocomposite with significantly lowered zeta potential (Cu5, -15.6 mV). Additionally, prolonging of reaction duration up to 60 min resulted in estimation of zeta potential with much lowered value (Cu4, -12.8 mV). This data is shown to be in harmony with all of the above-discussed data.

### 3.4 XRD and XPS results

**Fig. 5:**
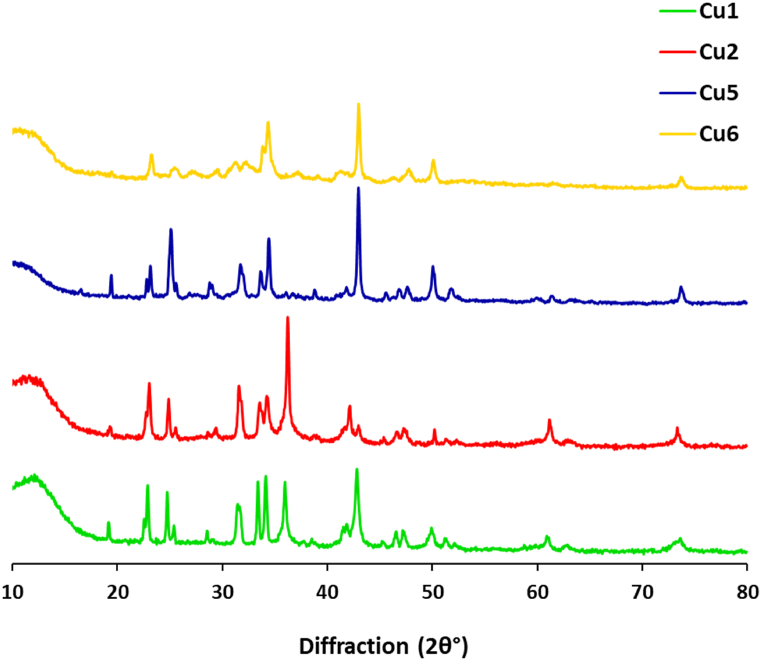
XRD diffraction for the prepared Cu@SH nanocomposite.

X-ray Diffraction data were represented in Fig. 5 where the effect of the different experimental conditions on the crystallinity degree for the as-synthesized Cu@SH nanocomposites was monitored. All of the examined samples (Cu1, Cu2, Cu5 and Cu6) exhibited three characteristic diffractions at 2θ° = 42.6°, 50.2° and 74.2°. These reported diffractions are indexed for (111), (200) and (220) planes, respectively. The diffractions agree with the standard diffractions for pure cubic CuNPs (JCPDS No. 040836) and with that mentioned for the face centered cubic (fcc) metallic copper (Cu^0^) [[46], [47], [48]]. The diffractions data supported that 10.13039/501100001838TEM observations and particle size results and further confirm the successful bio-fabrication of Cu@10.13039/100024172SH nanocomposites.

**Fig. 6:**
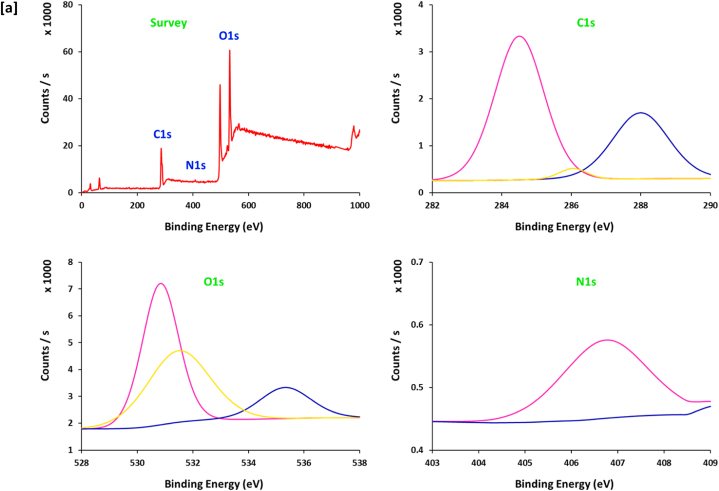

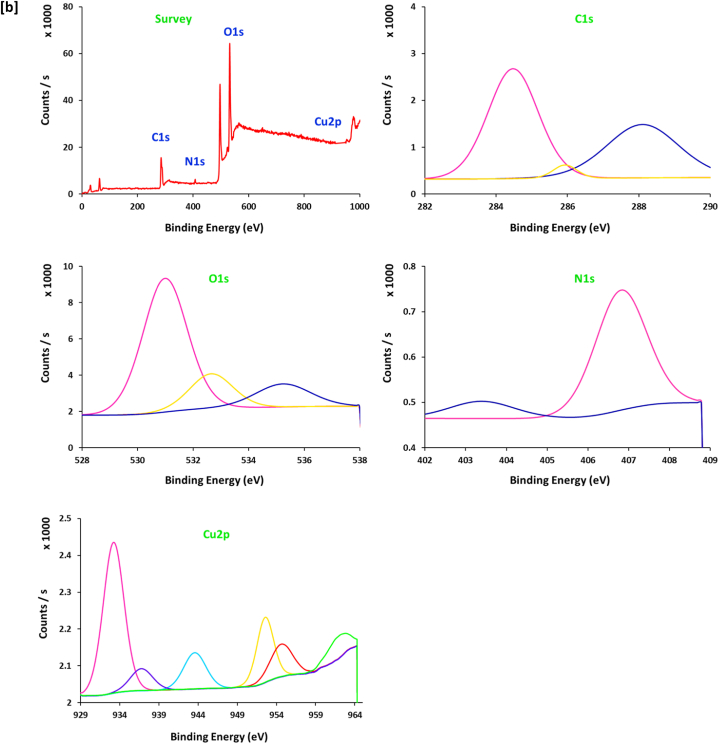

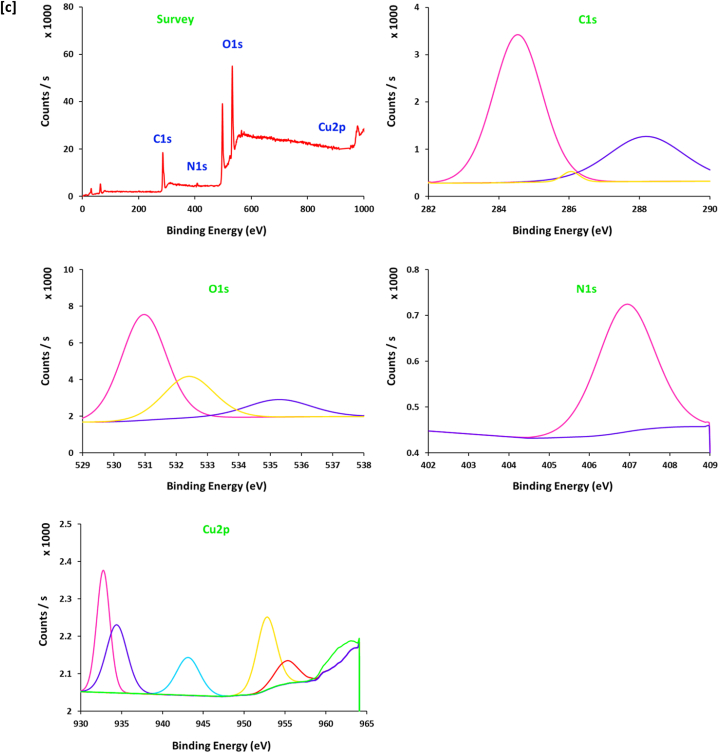
XPS spectra for the prepared Cu@SH nanocomposite; **[a]** SH, **[b]** Cu4, **[c]** Cu6.

The spectral data of XPS (Fig. 6) could support further confirmation for the electronic & chemical interaction between 10.13039/100024172SH ingredients and copper ions for reduction and stabilization [49], in addition to affirm the chemical composition of the prepared Cu@SH nanocomposites. Scanning the spectrum of Fig. 6a shows that, SH exhibits three characterized signals at the binding energy of "282–290 eV", "365–376 eV" and "528–537 eV", assigned for "C1s", N1s" and "O1s" respectively [50]. Signals of C1s were estimated at "285.2, 286.8 and 288.2 eV", are corresponding to "C-H, C-C and C-O″, respectively [[50], [51], [52]]. The three bands for O1s at "531.1 eV, 323.4 eV" and 535.4 eV, are referring to "O-H, C-O and O-Cu", respectively [[51], [52], [53]]. The spectral energy peaks for the Cu@SH nanocomposites are related to Cu2p ½ and Cu2p _3/2_. The main spectrum of Cu2p displays characteristic signals at 933.1 and 952.2 eV that are assigned for metallic copper (Cu^0^) and the small spectral peak at 936.3 and 953.8 eV corresponded to Cu(OH)_2_ [54,55]. This gives more confirmation for the successful clustering of Cu@SH nanocomposites.

### 3.5 Antimicrobial performance of Cu@SH nanocomposite

**Fig. 7:**
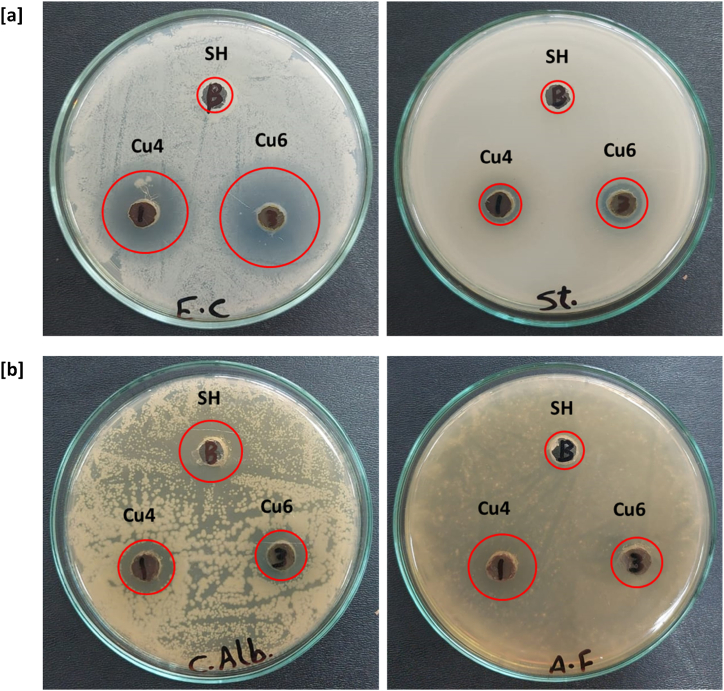
Digital photos for the antimicrobial activity for the prepared Cu@SH nanocomposite via inhibition zone test against gram-positive bacterial species (*Staphylococcus Aureus* ATCC-6538), gram-negative bacterial species (*Escherichia Coli* ATCC-25922) and two fungal strains namely (*Candida Albicans* ATCC-10231, Aspergillus Fumigatus ATCC-1022).; **[a]** bacteria, **[b]** fungi.

The antimicrobial performance of the synthesized Cu@SH nanocomposites was examined via in vitro examination against four different microbial strains, whereas the inhibition zone data were listed in Table 2. The tested samples exhibited a reasonable antimicrobial potentiality against the tested pathogenic species, and showed excellency in inhibitory impact against *" St. aureus", "E. coli", "C. Albicans"* and *"A. Fumigatus"* as shown in Fig. 7. Sider honey as a reference was exhibited with inhibition zone diameter of zero mm, zero mm, 15 mm and zero mm against the tested *E. Coli, St. aureus, C. Albicans and A. Fumigatus* strains, to affirm that SH as a reference shows non-antimicrobial potency. Whereas, Cu4 exhibited I.Z. diameter of 17 mm, 11 mm, 14 mm and 15 mm against *E. Coli, St. aureus, C. Albicans and A. Fumigatus* strains, respectively. The collected data show that, Cu6 exhibited the highest antimicrobial performance, with I.Z. diameter of 19 mm, 12 mm, 13 mm and 13 mm against *E. Coli, St. aureus, C. Albicans and A. Fumigatus* strains, respectively*.* So, it could be depicted that, compared to Cu4, the smaller size of Cu@SH nanocomposite ingrained in Cu6 sample caused superior antimicrobial impact. Meanwhile, the smaller size and more controllable geometry of nanocomposite is logically leading to an easier penetration of microbial followed by an inhibition of cell growth [56]. The obtained antibacterial and antifungal results were compared with that of tetracycline as conventional antibacterial drug and cyclohexamide as antifungal treatment, respectively. The synthesized Cu@SH nanocomposites showed very good antibacterial activity in comparison to tetracycline, especially for *E. Coli*. The synthesized Cu@SH nanocomposites exhibited quite good antifungal potency relative to cyclohexamide for both fungal strains (*C. Albicans* and *A. Fumigatus*). So, it could be concluded that, Cu@SH nanocomposites showed high affinity to be applicable as one of the therapeutic drugs for microbial infectious diseases and even for any of microbial associated health disorders.

**Table 2:** Antimicrobial activity (inhibition zone, mm) of the tested samples using the agar diffusion method.

**Sample**	**Bacteria**		**Fungi**	
	**G-Negative**	**G-Positive**		
	*Escherichia Coli* ATCC-25922	*Staphylococcus Aureus* ATCC-6538	*Candida Albicans* ATCC-10231	*Aspergillus Fumigatus* ATCC-1022
**SH**	0	0	15	0
**Cu4**	17	11	14	15
**Cu6**	19	12	13	13
**Tetracycline**	22	20	–	–
**Cyclohexamide**	–	–	18	18

### 3.6 Anticancer potentiality

**Fig. 8:**
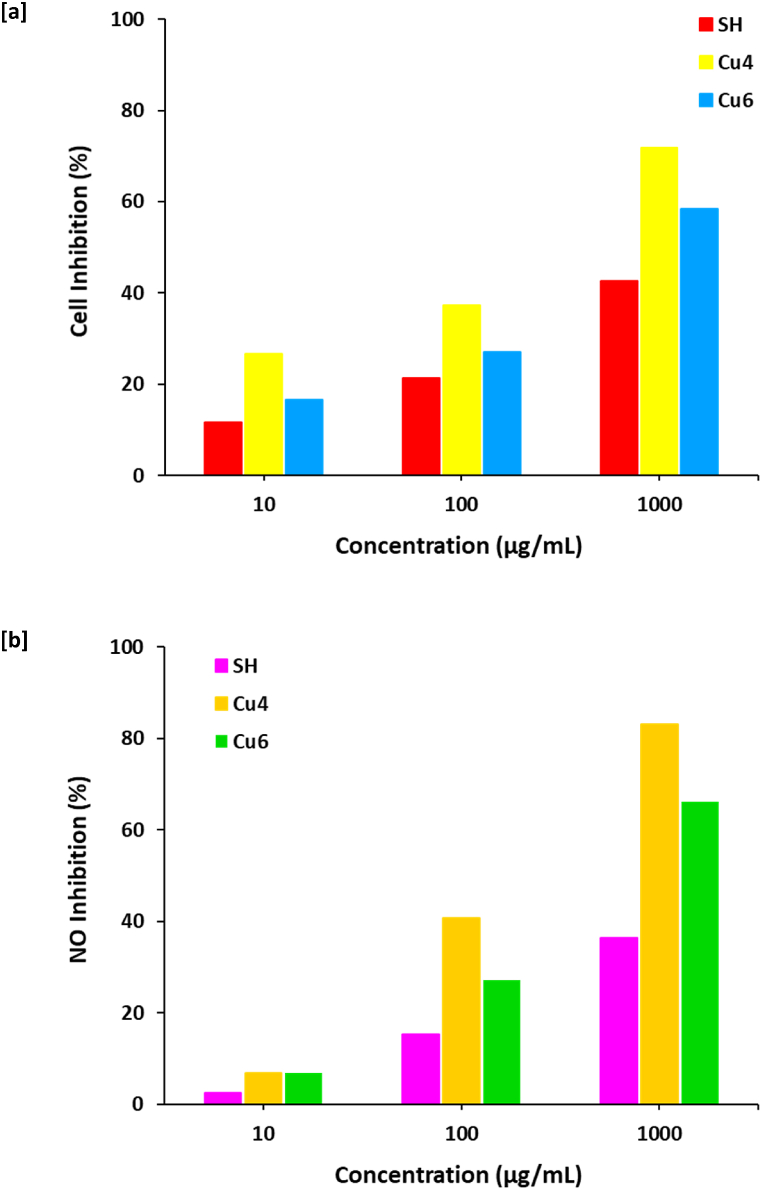
Bio-activity for the prepared Cu@SH nanocomposite; **[a]** anticancer and **[b]** anti-inflammatory.

The propensity of any anticancer drug for inhibition the growth of tumor cells is expressed as the cytotoxic effects, whereas, such drugs were suggested to act for the cell death via motivation of apoptosis (i.e., programmed cell death). In accordance to literature [57], apoptosis is ascribed as the most applicable mode of action for the anticancer reagents. Recently, different reports were interested in the investigation of the performance of different drugs that acted via the initiation of the apoptosis for malignant cells. Moreover, the activation of caspases is recognized as the main step for initiating and progressing of apoptosis. Caspases are endoproteases that act in the regulation of apoptosis signal networks and inflammation, by starting of the cellular deterioration (i.e., cyto-proteins degradation or DNA fragmentation) [58]. In accordance to literature [59], the anticancer potency and the anti-proliferative action of the prepared Cu-SH nanocomposites in the current approach can be elucidated as follows; the penetration of nanocomposite via the outer cell wall acts in activating of the caspases, as the as-synthesized nanocomposite in cytoplasm of the examined cells liberates ROS, to activate the caspases for direct necrosis. Meaning that, the smaller is the nanocomposite, the easier is the penetration via the outer cell wall prior to direct necrosis. Fig. 8a shows the relationship between the evaluated data of cell inhibition percent under the effect of Sider honey and Cu4 & Cu6 nanocomposites. The figured-out data obviously show that; compared to SH (1000 μg/mL, 43 %) and due to the insignificant difference in particle size for the as examined samples, both were shown with significantly high percentage of inhibition for cancer cells, the effect of Cu precursor concentration is consequently studied. Whereas, increment of nanocomposite concentration is reflected in estimation of higher percentage of cancer cell inhibition, as the nanocomposite that was prepared with higher concentration of copper precursor is exhibited with higher percentage of cancer cell inhibition. A 1000 μg/mL of Cu6 prepared with 5 mM/L of copper salt is exhibited with cell inhibition percentage of 62 %, while, Cu4 prepared with 10 mM/L of copper salt is shown with the highest anticancer performance (77 %). Premetrexed was used as standard chemotherapy for treatment of lung cancer. At the same concentration used (1000 μg/mL), the standard of premetrexed showed quite higher cancer cell inhibition (94 %). However, the applied nanocomposite exhibited good cell inhibition. This can declare that; the anti-cancer performance of the prepared nanocomposite is mainly correlated to its content of copper. So, it could be affirmed that, the prepared nanocomposite can be successfully exploited as anti-cancer drug.

### 3.7 Anti-inflammation performance

The main effect of inflammation is the decomposition of cell proteins [60]. Whereas, the decomposed proteins act in the extensive motivation of immune cells in the inflammation site, leading to the dramatical transformation of "acute inflammation" to "chronic inflammation" [60,61]. Moreover, "Tumorigenesis" (the progress in the growth of cancer cells), is resulted from "chronic inflammation" [62]. Transforming of "acute inflammation" to "chronic inflammation" occurs when the mechanism of controlling "acute inflammation" is inefficiently performed and the clearance of inflammation mediators is not proceeded [63]. Subsequently, the inflammation mediators are continuously increasing, leading to the generation of ROS (such as O_2_^**.**^, OH^**.**^, ^1^O_2_, and H_2_O_2_) and RNS as nitrogen reactive species (such as NO^**.**^, ONOO^−^) that mainly act in the decomposition of nucleic acids [63,64]. Excessive contents of ROS & RNS leaded to the oxidation of biomolecules and decomposition of proteins and genes, reflected in the transformation of "acute inflammation" to "chronic inflammation" [65]. So, it could be declared that, the action of anti-inflammatory reagents is mainly the acceleration of ROS and RNS sweeping to inhibit the inflammation process.

In the current work, Griess assay was performed for studying the propensity of the prepared nanocomposites as anti-inflammatory reagents. Herein, the anti-inflammation action was approved via the estimation of the inhibition percentage of nitrous oxide radicals (NO^**.**^) as one of RNS that is responsible for transforming "acute inflammation" to "chronic inflammation". Meaning that, higher is the anti-inflammation performance, lower is the percentage of NO^**.**^ (NO %). Fig. 8b shows the relationship between the concentration of the synthesized nanocomposites and NO % to declare that, the higher the concentration of the prepared nanocomposites, the lower the NO %, as estimated to be 89 % & 18 % for Cu4 with concentration of 10 & 1000 μg/mL, respectively. Although, both of the examined nanocomposites were shown with high efficiency of anti-inflammatory performance, however, the data showed the supremacy of the nanocomposite prepared with higher concentration of copper salt (Cu4, 1000 μg/mL) to be estimated with the lowest NO % of 18 %, whereas, nanocomposite prepared with lower concentration of copper salt (Cu6, 1000 μg/mL) is exhibited with higher NO % of 68 %. Naproxen as a common anti-inflammatory drug was used as the control. The NO% inhibition ratio was quite higher (98 %) for the standard agent (Naproxen), however, Cu@SH nanocomposite showed effective antifungal activity at high concentration that reflect their possibility for anti-inflammation diseases.

## 4 Conclusion

Green synthesis of nanostructures promotes the successive clustering of nanostructures in environmentally safe pathway for both human beings and the surrounding environment. Through green nano-synthesis, no toxic chemical byproducts are discharged to the surrounding environment. Currently, the point of novelty in the demonstrated approach is to investigate the green affinity of Egyptian sidr honey (SH) to act as a substrate for successive clustering of copper-based nanocomposite, abbreviated as Cu@SH, in order to be applicable as antimicrobial/therapeutic drug. SH played the dual role as reducer and stabilizer, without adding any of extra chemical ingredients. NMR spectral mapping data were examined in order to confirm the composition of Cu@SH nanocomposite. The successive clustering of Cu@SH nanocomposites was monitored via visual color change, UV–visible spectral data, Transmission Electron Microscope (TEM), Zeta sizer, X-ray diffraction (XRD) and X-ray photoelectron spectroscopy (XPS). The obtained Cu@SH nanocomposites were examined as antimicrobial therapeutic drug against different microbial pathogenic strains. The overall results affirmed the under the optimal experimental conditions, spherical shaped Cu@SH nanocomposites with quite small particle size were successfully clustered. The Cu@SH nanocomposites show superior antimicrobial action against all of the examined strains. Moreover, the prepared nanocomposites showed supremacy as anticancer and anti-inflammatory drugs. In summarization, Cu@SH nanocomposites showed high affinity to be concurrently applicable as therapeutic drugs for the microbial infectious, cancer and inflammation diseases.

## CRediT authorship contribution statement

**Nehal Eid:** Methodology, Investigation, Formal analysis, Data curation, Conceptualization. **Hesham R. El-Seedi:** Visualization, Validation, Supervision, Data curation, Conceptualization. **Hanan B. Ahmed:** Writing – review & editing, Writing – original draft, Visualization, Validation, Data curation. **Hossam E. Emam:** Writing – review & editing, Visualization, Validation, Supervision, Investigation, Conceptualization.

## Ethical approval

Not applicable.

## The data availability statement

All data generated or analyzed during this study are included in this published article.

## Funding

Not applied.

## Declaration of competing interest

Authors declare that they have no conflict of interest.
