# Supplementary material for: Bio-fabrication of biologically active copper nanocomposite
Source: Heliyon. 2024 Nov 9;10(22):e40202. doi: 10.1016/j.heliyon.2024.e40202 (PMC11585709; doi:10.1016/j.heliyon.2024.e40202)
Supplement: Multimedia component 1 [file mmc1.docx]

**Supplementary file**

**Figure S1:** NMR spectra for Cu@SH nanocomposite; **[a, b]** ^1^HNMR, **[c, d]** ^13^CNMR, **[a, c]** Cu4 and **[b, d]** Cu6.

| **[a]** | **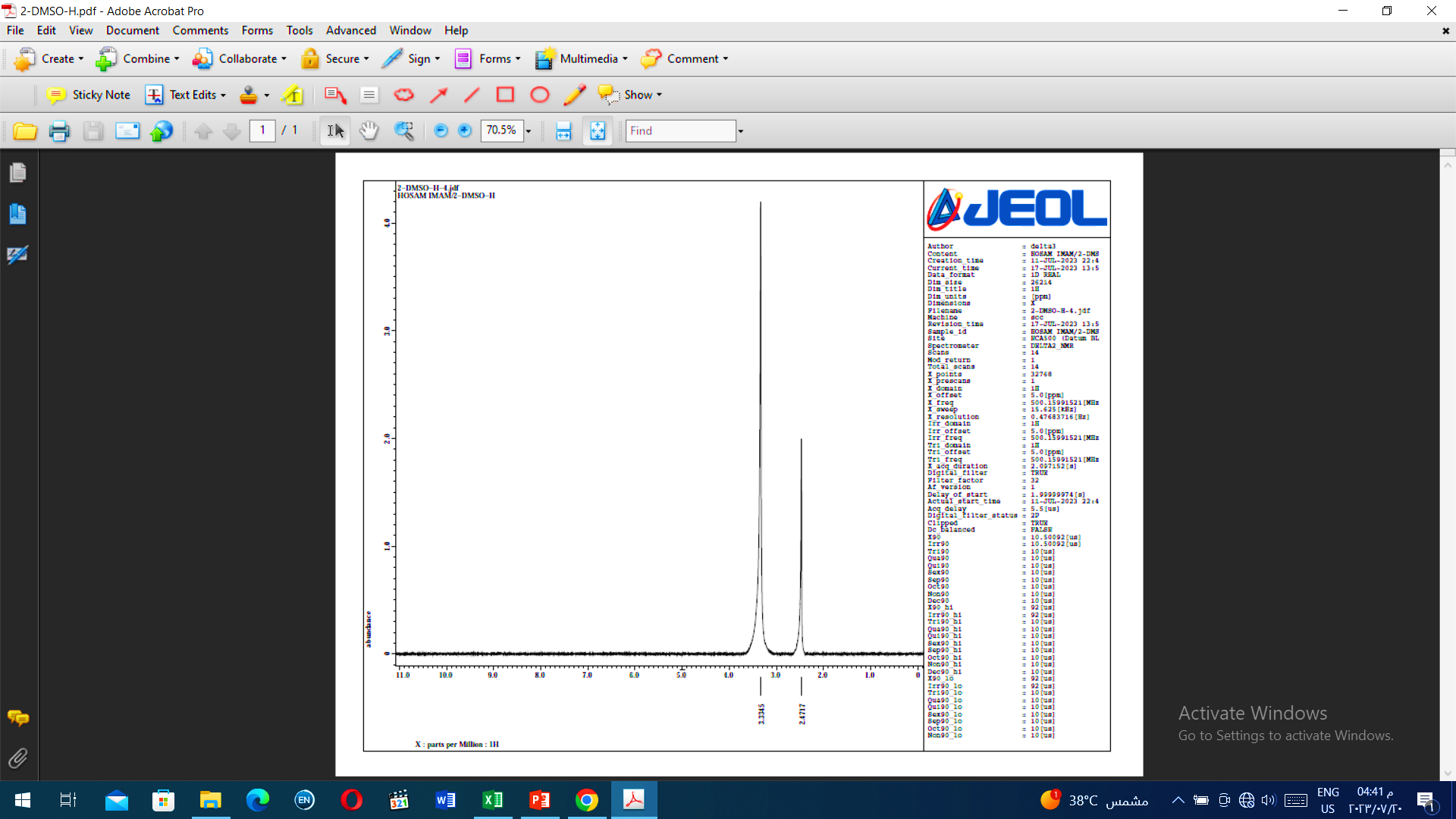** |
| --- | --- |
| **[b]** | **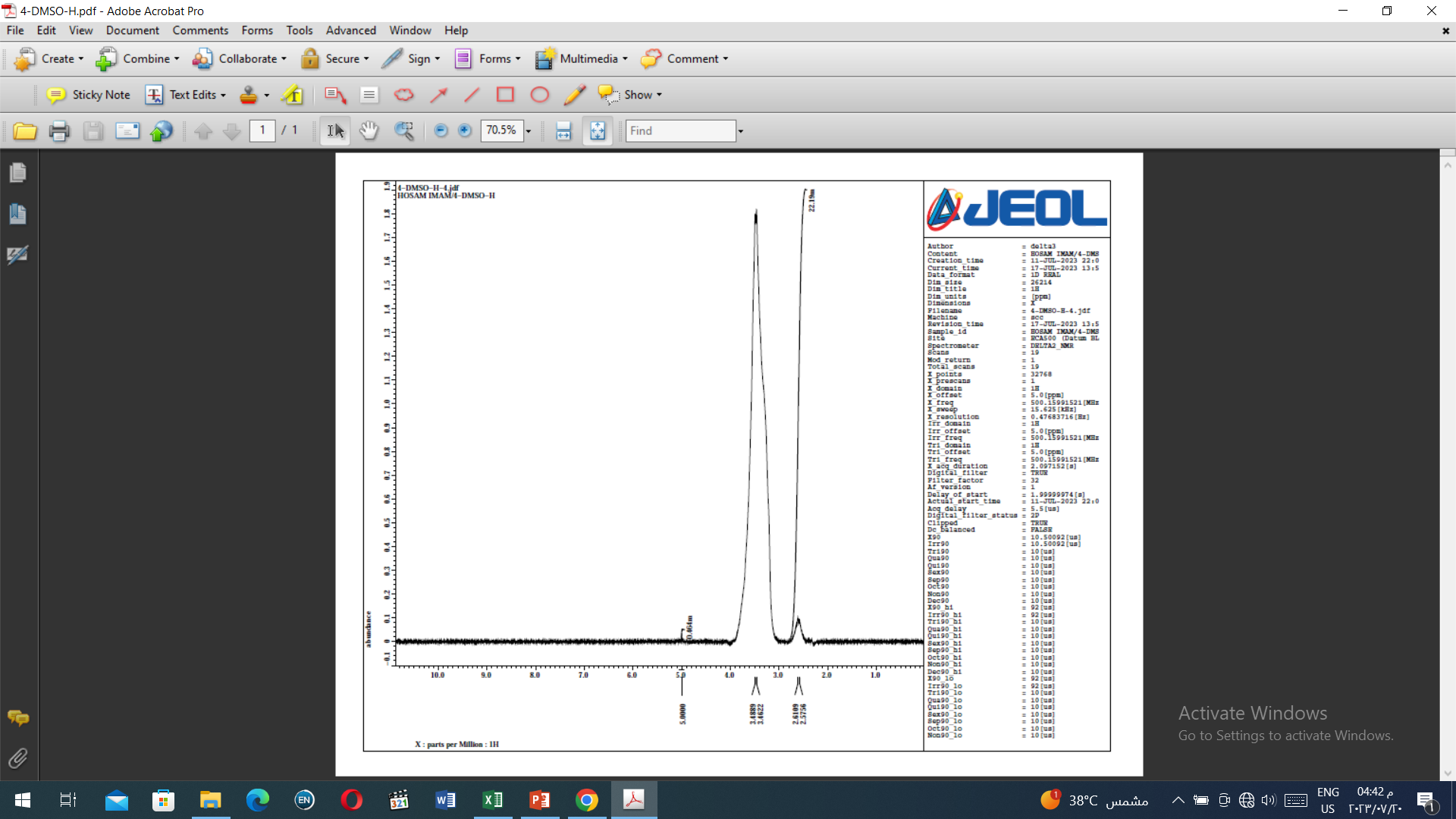** |
| **[c]** | **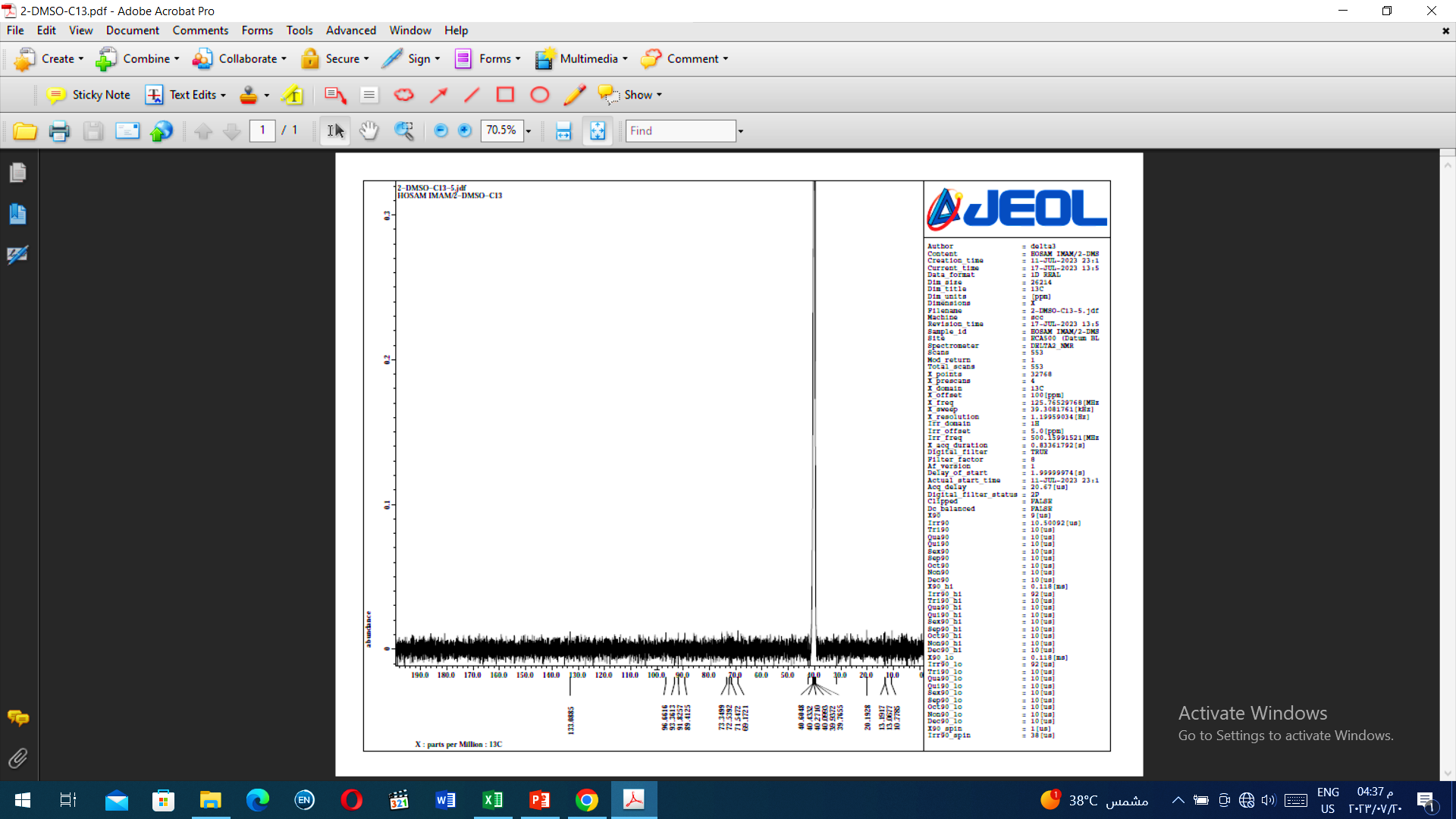** |
| **[d]** | **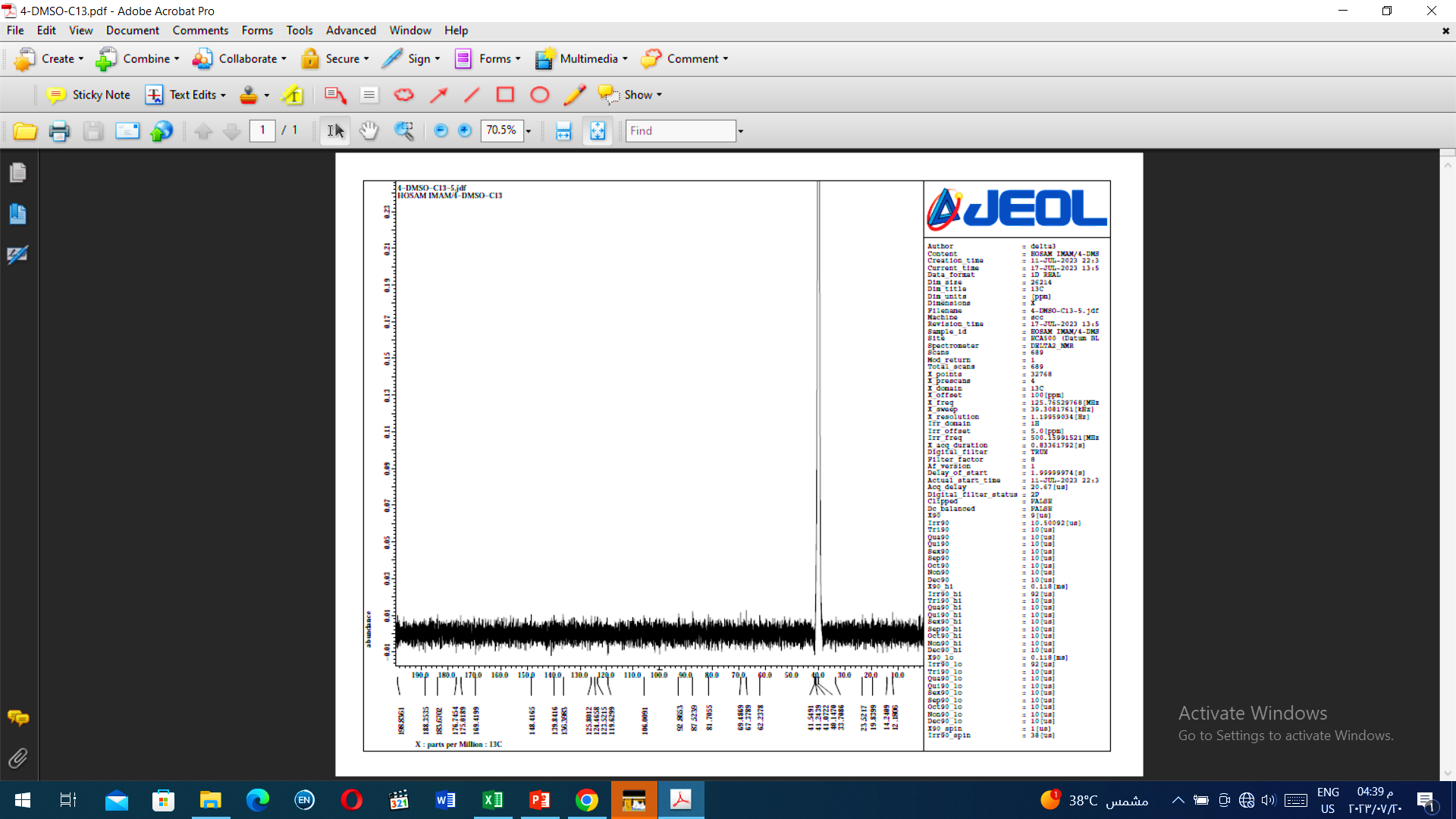** |
